# Supplementary material for: Blocking the shikimate pathway amplifies the impact of carvacrol on biofilm formation in Candida albicans
Source: Microbiol Spectr. 2025 Feb 7;13(3):e02754-24. doi: 10.1128/spectrum.02754-24 (PMC11878086; doi:10.1128/spectrum.02754-24)
Supplement: Figures S1 to S4 — Growth curves, gene essentiality screen, full PABA rescue experiments, carvacrol impact on mycelial growth. [file spectrum.02754-24-s0001.pdf]

Blocking the shikimate pathway amplifies the impact of carvacrol on biofilm formation in *Candida albicans*

Ali Molaeitabari and Tanya E. S. Dahms

Supplemental Material

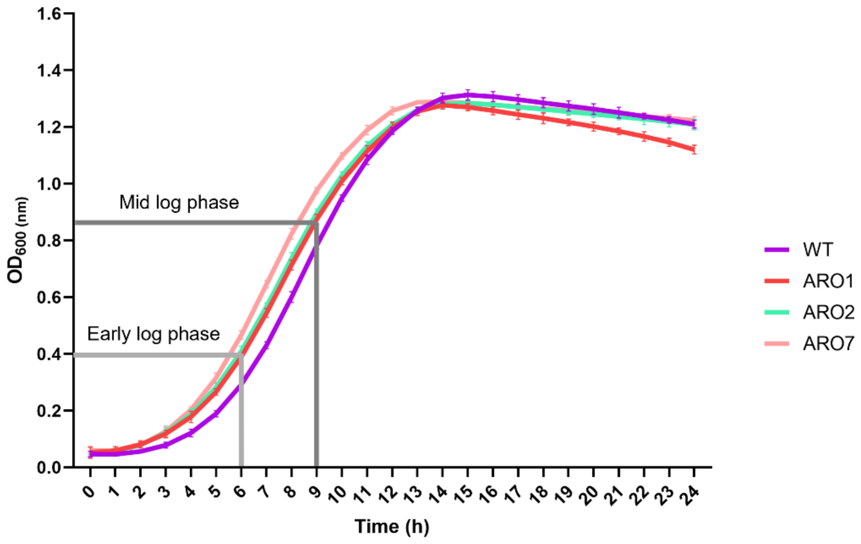

Figure S1. Growth curve of *C. albicans ARO1*, *ARO2* and *ARO7* mutants and WT strain. Optical density (600 nm) of *C. albicans* grown at 30 °C for 24 h.

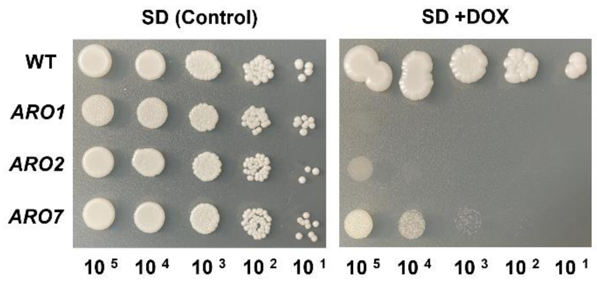

Figure S2. Gene essentiality screening confirms that the *ARO1* gene is essential for cell viability. Representative cells at 10^5, 10^4, 10^3, 10^2 and 10^1 CFU were spotted onto SD solid medium with or without DOX and assessed after 2 d incubation at 30 °C.

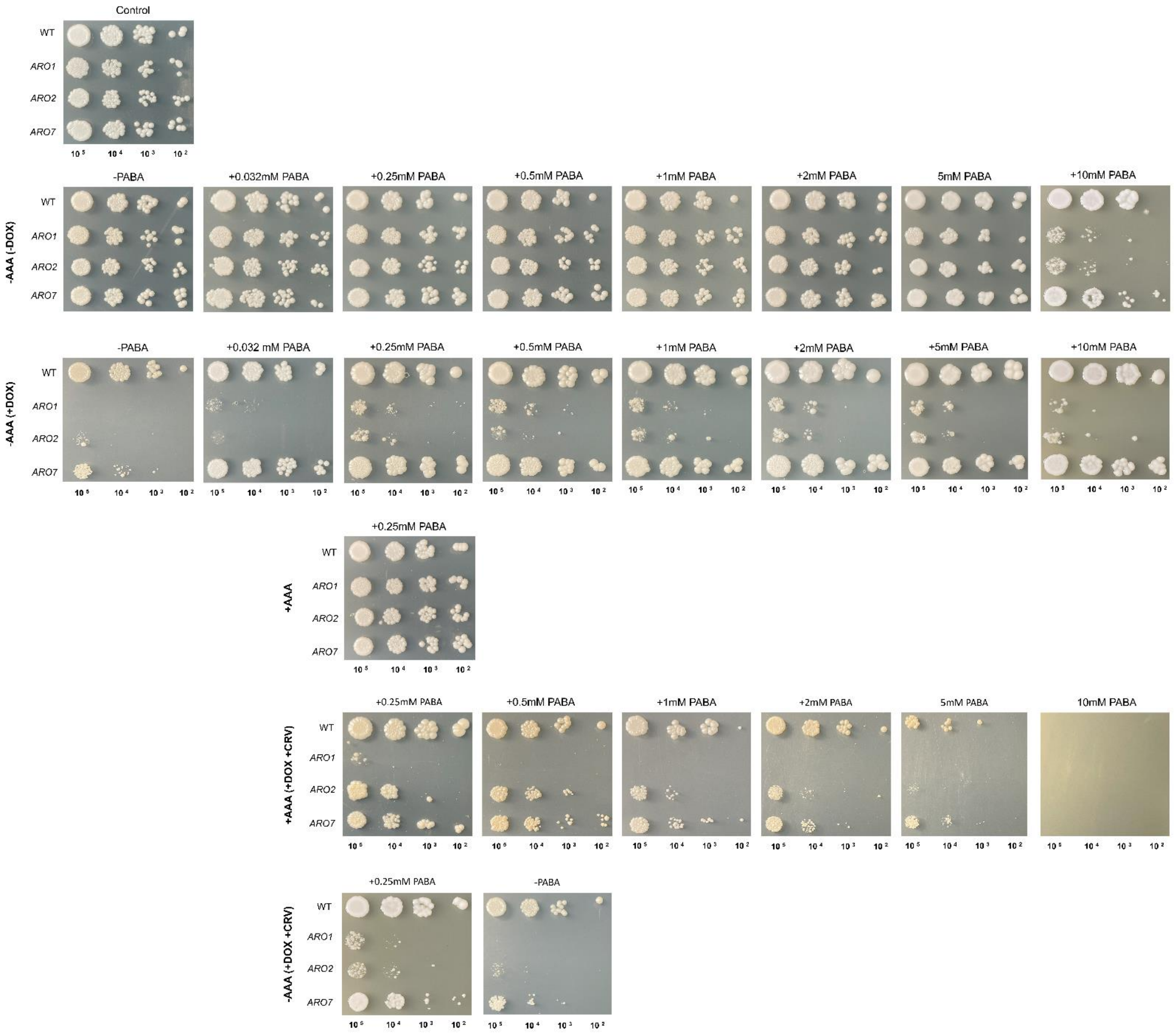

Figure S3. Repressed mutants are partially rescued in the presence of 0.25 mM PABA. The WT strain and *ARO1*, *ARO2* and *ARO7* mutant overnight cultures were serially diluted (10^5, 10^4, 10^3 and 10^2 CFU) and spotted on SC medium supplemented with different concentrations of PABA with/without aromatic amino acids, either in the presence or absence of DOX or carvacrol at sublethal levels (1/2 MIC). The plates were incubated at 30 °C for 2 days and then photographed. CRV: carvacrol; AAA: aromatic amino acid; PABA: para-aminobenzoic acid

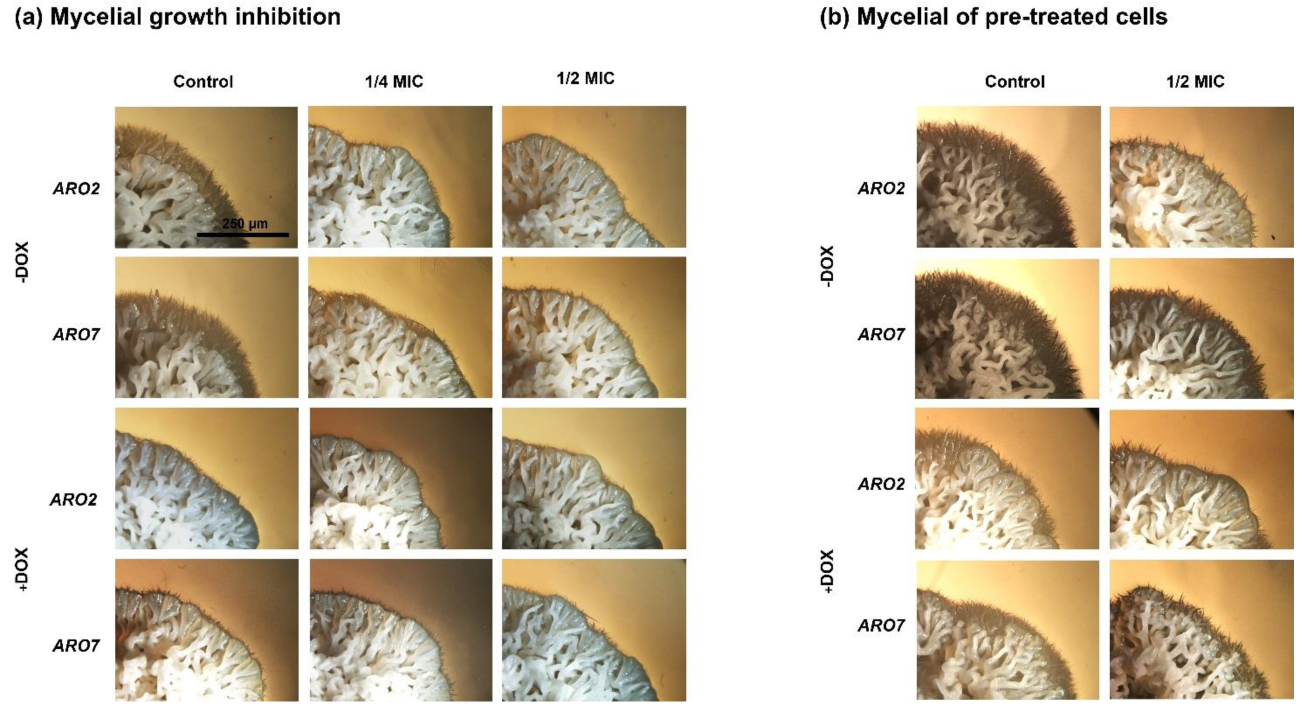

Figure S4. Carvacrol impacts *C. albicans ARO2* and *ARO7* mycelial growth. Representative stereoscopic bright-field images of the *ARO2* and *ARO7* mutants on spider media agar plates show colony morphology with a) constant exposure to carvacrol (6 d) b) 4 h pre-treatment with carvacrol, followed by 6 d incubation in the absence of carvacrol. Scale bar = 250 µm, applicable to all images.
